# Clinical Significance of Lymph Node Micrometastasis in pN0 Gastric Cancer Patients

**DOI:** 10.1155/2021/6854646

**Published:** 2021-03-03

**Authors:** Yu Li, Dongsheng Wang, Yi Li, Xiaodong Liu, Dong Chen, Chentong Yuan, Yanbing Zhou

**Affiliations:** Department of Gastrointestinal Surgery, The Affiliated Hospital of Qingdao University, Qingdao, Shandong, China

## Abstract

**Purpose:**

To investigate the relationship between lymph node micrometastasis (LNMM) and clinicopathological factors and to evaluate the prognostic effects of LNMM in pN0 gastric cancer (GC) patients.

**Methods:**

One hundred and seventy-two GC patients who received radical gastrectomy with D2 lymph node dissection were enrolled in the present study. 1371 negative lymph nodes from level 2 station confirmed by pathology were examined. The LNMM was diagnosed by telomeric repeat amplification protocol/enzyme-linked immunosorbent assay (TRAP-ELISA). The relationship between clinicopathological factors and LNMM was investigated by multivariate analysis. Survival analysis was performed to evaluate the effects of LNMM on prognosis.

**Results:**

LNMM was detected in 423 lymph nodes from 72 patients. The results showed that invasion depth (OR = 3.755, *P* = 0.004), TNM staging (OR = 3.152, *P* = 0.002), lymphatic invasion (OR = 2.178, *P* = 0.009), and tumor differentiation (OR = 1.266, *P* = 0.013) were independent risk factors associated with LNMM. Survival analysis showed that patients with LNMM had significantly worse 5-year survival compared with those without LNMM (42% vs. 76.4%, *P* < 0.05). Multivariate analysis demonstrated that LNMM, tumor size, Lauren type, invasion depth, and lymphatic invasion (*P* < 0.05) were independently factors associated with 5-year survival.

**Conclusions:**

The findings showed that tumor invasion depth, TNM staging, lymphatic invasion, and tumor differentiation were independent risk factors associated with LNMM occurrence. Moreover, LNMM is a clinically negative prognostic factor in pN0 GC patients.

## 1. Introduction

Gastric cancer (GC) is the fifth most frequently diagnosed cancer and the third leading cause of cancer death worldwide, which is responsible for over 1,000,000 new cases and an estimated 783,000 deaths in 2018 [1]. Despite the progress in surgical techniques and oncologic therapies, the prognosis of GC patients is disappointing, with a 5-year overall survival rate remaining at 28% in most areas of the world, except in Japan, where a 5-year survival rate is up to 70% [2]. Lymph node metastasis is currently considered to be one of the most significant prognostic factors in GC patients. Therefore, radical gastrectomy with D2 lymph node dissection is recognized as the standard surgical treatment of GC in East Asia [3].

However, despite curative resection of primary tumor and lymphadenectomy, some patients with histologically node-negative GC still die of local or distant tumor recurrence [4–6]. Previous studies suggest that lymph node micrometastasis (LNMM) may be one of the key causative factor for GC recurrence [7]. According to the 8th edition of American Joint Committee on Cancer (AJCC) staging system, micrometastasis (MM) is defined as tumor deposits greater than 0.2 mm but less than 2.0 mm in largest dimension that is negative by conventional histological examinations, but positive by advanced diagnostic tools [8]. Although prior studies have attempted to clarify the relationship between LNMM and clinicopathological features and to explore the prognostic value of LNMM in GC patients [5, 9, 10], the clinical significance of MM remains controversial currently.

With the rapid development of molecular biology technology, such as immunohistochemistry (IHC) and reverse transcription-polymerase chain reaction (RT-PCR), the detection rate of MM has increased significantly [11]. Telomerase is a specialized cellular reverse transcriptase that uses its RNA (Ribonucleic acid) template to elongate the telomere by adding G-rich telomeric repeats to its terminal 3′ overhang and is strongly suppressed in human somatic cells; however, robust telomerase activity is seen in highly proliferative tissues as well as in cancer cells [12]. Several studies have indicated that telomerase reactivation plays an important role in gastric carcinogenesis, and telomerase activity can be used as a marker for neoplastic transformation in GC [13]. Hu et al. reported that telomeric repeat amplification protocol/enzyme-linked immunosorbent assay (TRAP-ELISA) was significantly more sensitive than cytology or CA125 assay in detecting early peritoneal metastasis [14]. However, to the best of our knowledge, few studies have used this technique to detect LNMM in GC patients.

Therefore, the aim of this study is to investigate the incidence of LNMM in pN0 GC patients using TRAP-ELISA, to explore the relationship between LNMM and clinicopathological factors and to evaluate the prognostic value of LNMM in 5-year survival.

## 2. Materials and Methods

### 2.1. Patients

**Figure 1:**
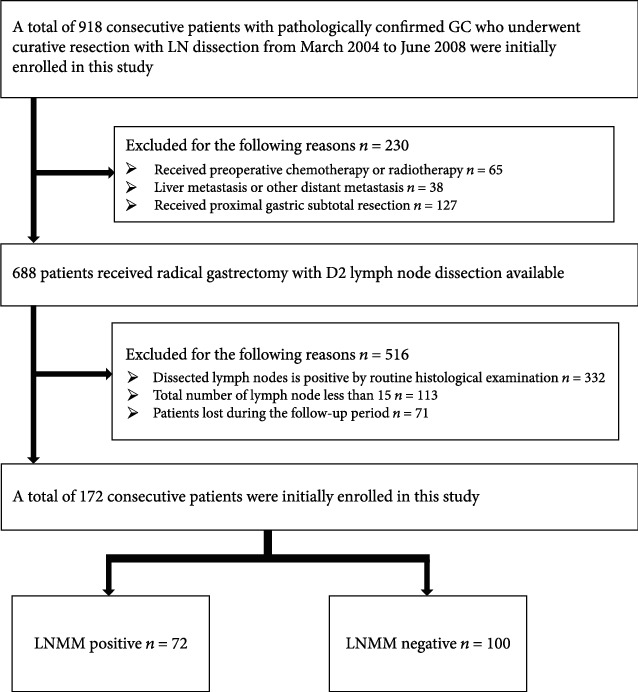
Recruitment pathway for patients in this study.

Ethical approval was obtained from the medical ethics committee of the Affiliated Hospital of Qingdao University for this retrospective study. 918 consecutive patients diagnosed of GC who received gastrectomy with D2 lymph node dissection from March 2004 to June 2008 in the Affiliated Hospital of Qingdao University were chosen for this study. All the patients were admitted according to the patient recruitment pathway using the inclusion and exclusion criteria described in Figure 1. A total of 172 patients were incorporated into our study finally.

Clinical data, such as age, gender, and surgical approach, were collected by reviewing medical records. Tumor size, depth of invasion, tumor differentiation, Lauren classification, lymphatic infiltration, and neural invasion were obtained from pathological reports directly. TNM staging was established postoperatively by surgical oncologists and pathologists according to the American Joint Committee on Cancer (AJCC) TNM staging system, the 8th edition [8].

### 2.2. Specimens Collection

The dissected lymph nodes were classified according to the Japanese Classification of Gastric Carcinoma, 3^rd^ English edition [15], and the No. 7, 8a, 9, 11p, 12a lymph nodes were defined as the level 2 station lymph nodes. Macroscopic lymph nodes from GC specimens were obtained within 15 to 30 minutes after dissection during the operation. Each fresh lymph node specimen was divided equally into two halves. One half was prepared for routine histological examinations, and the other half was immediately frozen in liquid nitrogen for TRAP-ELISA.

### 2.3. TRAP-ELISA

Frozen samples were mixed with precooled cell lysis reagent, homogenized and incubated on ice for 30 min, and then centrifuged at 16000 r/min. The supernatant was collected for the TRAP assay using TRAP reaction kit (Medac, Wedel, Germany). For each sample to be tested, a 50 *μ*l reaction mixture containing taq DNA polymerase, primers (CS5′ CCCTTACCCTTACCCTTACCCCA 3′; TS 5′AATCCGTCGTCG AGCCAGAGTT 3′), and 3 *μ*g protein extraction from the supernatant was prepared. Each reaction mixture was subjected to primer extension at 25°C for 20 min, telomerase inactivation at 94°C for 5 min followed with incubation at 90°C for 3 min. Then, each reaction mixture was subjected to 35 PCR cycles of denaturation at 50°C for 30 seconds and extension at 72°C for 90 s, followed by a final cycle of extension at 72°C for 90 s. Finally, the reaction mixtures were incubated at 4°C for reaction termination.

ELISA was performed as described previously [14]. Briefly, 5 *μ*l amplification product was mixed with 20 *μ*l denaturation reagent and incubated at room temperature for 10 min. Then, 255 *μ*l of hybridization buffer was added into the mixture. 100 *μ*l/well of the mixture was added into a precoated microtiter plate (MTP) module and incubated at 37°C on a shaker (300 rpm) for 2 h. After adding 100 *μ*l/well anti-digoxigenin-peroxidase (DIG–POD) working solution, the MTP module was incubated at room temperature for 30 min while shaking at 300 rpm. The reaction mixture was then removed, and 100 *μ*l anti-DIG–POD working solution was added per well and incubated at room temperature for 30 min while shaking at 300 rpm. The wells were then washed 5 times with a washing buffer for a minimum of 30 s each. After the final wash, 100 *μ*l/well tetramethylbenzidine substrate solution was added and incubated at room temperature for 10–20 min while shaking at 300 rpm. The absorbance value for each sample was calculated as Absorbance (units) = A450 − A690. Lysis supernatant inactivated at 85°C for 10 min was used as a negative control. As positive control, we used an extract of telomerase positive embryonic kidney cell line 293, provided with the kit. Negative controls were prepared from positive controls, considering that telomerase essentially requires integrity of its internal RNA component as a template for the addition of the telomeric repeat sequences to the telomerase-specific primer [16].

### 2.4. Follow-Up and Statistical Analysis

Patients were followed up for disease recurrence and long-term survival until the end of the study (Dec 25, 2014) or disease recurrence or death. Association between patients' clinical characteristics and LNMM was analyzed by multivariate analysis using the Cox proportional hazards model. Variables were analyzed using the *χ*^2^ test or Fisher's exact test. The Kaplan-Meier method was used to estimate overall survival. Prognostic factors for 5-year survival were analyzed using Cox proportional hazards regression model. *P* < 0.05 was considered statistically significant. Statistical analysis was performed using SPSS version 18.0 for Windows (SPSS, Chicago, Illinois, USA).

## 3. Results

### 3.1. Patients

From March 2004 to June 2008, a total of 172 patients with node-negative GC were enrolled in our study. Among the 172 patients, the mean age was 57.36 ± 12.13 years. All of the patients received radical gastrectomy (119 patients with distal gastrectomy and 53 patients with total gastrectomy) and D2 lymphadenectomy. A total of 4125 negative lymph nodes were obtained from 172 patients, among which 1371 lymph nodes were from level 2 station, and the average number of resected lymph nodes was 23.84 ± 9.04. Based on the TRAP–ELISA analysis, 423 level 2 station lymph nodes from 72 patients (41.86%72/172) had LNMM, including 8 lymph nodes from two early gastric cancer patients.

### 3.2. Clinicopathological Factors Associated with LNMM

Associations between clinicopathological characteristics and LNMM are shown in Table 1. The following clinicopathological variables were significantly associated with LNMM: tumor size, tumor differentiation, the depth of tumor invasion, lymphatic infiltration, Lauren classification, and TNM staging. As the depth of tumor extends, the rate of LNMM also increases. LNMM was found in 10.53% (2/19), 30.77% (8/26), 45.54% (46/101), and 61.54% (16/26) of patients with T1 (mucosa and submucosa), T2 (muscularis propria), T3 (subserosa), and T4 (serosal layer or beyond) depth invasion, respectively. Multivariate analysis showed that tumor differentiation (OR = 1.266, 95% CI 4.274-37.037, *P* = 0.013), lymphatic infiltration (OR = 2.178, 95% CI 1.635-6.327, *P* = 0.009), the depth of tumor invasion (OR = 3.755, 95% CI 1.716-8.218, *P* = 0.004), and TNM staging (OR = 3.152, 95% CI 1.547-7.589, *P* = 0.002) were independent risk factors associated with LNMM (Table 2).

### 3.3. Overall Survival

**Figure 2:**
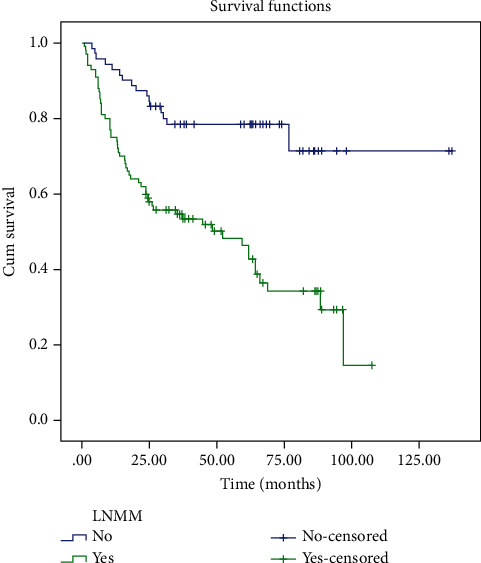
Kaplan-Meier disease survival curve according to the presence of LNMM. 5-year overall survival (OS) rate was significantly longer in patients without LNMM vs. with LNMM (76.4% vs. 42%, *P* < 0.001).

Patients were followed up for long-term survival (Figure 2). The median follow-up time was 37.5 (range 1.2–137.0) months. Fourteen patients were lost during follow-up, and the follow-up rate was 91.9%. Seventy-five patients died due to recurrent gastric carcinoma. The 1-, 3-, 5-year overall survival rates were 75%, 64%, and 56%, respectively. Five-year overall survival (OS) rate was significantly longer in patients without LNMM vs. with LNMM (76.4% vs. 42%, *P* < 0.001).

Univariate analysis demonstrated that tumor size (*P* = 0.01), tumor differentiation (*P* < 0.01), the depth of tumor invasion (*P* = 0.01), lymphatic infiltration, Lauren classification (*P* = 0.01), TNM staging (*P* = 0.01), and LNMM (*P* = 0.01) were significantly associated with 5-year overall survival (Table 3). Additional multivariate analysis showed that level 2 station LNMM (HR = 2.476, 95% CI 1.415-4.334, *P* = 0.001), the depth of tumor invasion (HR = 1.418, 95% CI 1.060–1.897, *P* = 0.019), lymphatic infiltration (HR = 2.893, 95% CI 1.697-4.934, *P* = 0.00), Lauren classification (HR = 0.209, 95% CI 1.083-1.527, *P* = 0.001), tumor size (HR = 1.978, 95% CI 1.260-3.106, *P* = 0.003), and TNM staging (HR = 1.375, 95% CI 1.024-1.846, *P* = 0.034) were independent risk factors of 5-year survival (Table 4). Further analysis showed that LNMM was an independent prognostic indicator for patients with histologically node-negative T1-T3 GC. However, in patients with T4 disease, the prognostic value of LNMM was not significant (*P* = 0.568).

## 4. Discussion

In the present study, we found a higher LNMM rate (72/172, 41.86%) in pN0 GC patients. The multivariate logistic analysis showed that the depth of invasion, TNM staging, lymphatic infiltration, and tumor differentiation were independent risk factors associated with LNMM. Moreover, Cox proportional hazards regression mode demonstrated that LNMM, tumor size, Lauren type, invasion depth, and lymphatic infiltration were independent prognostic factors for long-term survival. Furthermore, our results showed that LNMM had a negative influence on 5-year survival in GC patients (76.4% vs. 42%, *P* < 0.001).

The detection rate of LNMM shows a great difference depending on applied methods and the stage of GC. Maehara et al. showed that the incidence of LNMM ranged from 4.8% to 36% in the early-stage GC patients with histologically node-negative [5]. Fukagawa et al. reported 23.5% of LNMM was observed in 34 node-negative GC patients with pT1 tumor by IHC [17]. Yasuda et al. demonstrated that LNMM was detected in 73 nodes (4%) and 20 patients (31%) with pT2 and pT3 tumors by IHC [18]. The incidence of LNMM is also closely related to the clinicopathological characteristics of GC patients. The previous study with advanced GC reported that the incidence of MM in lymph nodes increases in proportion to tumor size and the depth of invasion [10]. In our study, the identification rate of LNMM (72/172, 41.9%) was much higher than the previous studies. Furthermore, we found that with the tumor depth invading, MM occurrence rate corresponding increased (10.53% (2/19) T1, 30.77% (8/26) T2, 45.54% (46/101) T3, and 61.54% (16/26) T4). The main reason for explaining this higher result is that advanced stage GC patients occupy the majority of our data. In these patients, the depth of invasion, changes of lymph nodes, and lymphatic invasion may all increase the risk of tumor emboli and thereby increased the risk for LNMM [19, 20]. Besides the depth of invasion, we also revealed that tumor differentiation, lymphatic infiltration, and TNM staging were independent risk factors for LNMM.

In clinical, lymph node metastasis is considered to be a most reliable prognostic factor that has been widely applied in the TNM staging system to estimate prognosis and guide clinical decision-making. However, some researchers reported that even after undergoing curative surgery and conventional postoperative pN0 diagnosis does not guarantee against recurrence [21]. Several studies have demonstrated that the presence of LNMM has been identified as an independent risk factor associated with poor outcome in several malignancies including breast [22], colon [23], esophagus [24], gallbladder [25], and lung cancers [26]. Nakajo et al. reported that LNMM was correlated with a significantly lower survival rate in GC patients with T1 or T2 tumors [27]. Cai et al. found a significant association between LNMM and poor prognosis in patients with T3 GC patients [28]. Although most of studies have shown that LNMM is associated with poor prognosis in GC patients, some researchers still have different findings. Fukagawa et al. showed that the presence of LNMM did not affect survival in a large number of T2 GC patients [17]. In our previous meta-analysis, we performed 18 relevant studies, and the results demonstrated that LNMM had an intimate, significant relationship with a higher risk of disease recurrence and worse survival in GC patients [29]. In the present study, our results indicated that the level 2 station LNMM was indeed associated with poor survival in GC patients. Furthermore, our investigation revealed that LNMM is an independent prognostic indicator for pN0 GC patients with T1-T3. However, it has little prognostic value in T4 patients. The probable reason is that the prognostic significance of LNMM is compromised by peritoneal recurrences which frequently encountered in T4 tumors. Infiltrative tumors have a strong tendency to develop peritoneal seeding when they evolve into T4 tumors. Therefore, it is challenging to confirm the prognostic significance of LNMM in T4 GC patients.

Due to the importance of LNMM in the clinical, how to choose an accurate and sensitive detection tool of LNMM is crucial for disease management and prognosis prediction of GC patients. With the rapid development of molecular biology technology, numerous LNMM detection methods have emerged recently. The IHC staining with AE1/AE3 and TTF-1 is a classic method of detecting LNMM [30]. However, the IHC method was arduous because it requires the remarkable labor of preparing slides and allows senior pathologists to search for tiny tumor cells under the microscope. TRAP-ELISA is a new method with high sensitivity and accuracy for detecting MM. Hu et al. assessed telomerase activity in tumor samples and peritoneal washings from 46 GC patients, and the results showed that telomerase activity was detected in 89.1% of the GC tissue specimens and 47.8% of the peritoneal washing sample, which is much higher than those obtained by conventional cytological examinations [14]. In addition, they also revealed that telomerase activity was correlated with tumor grades, depth of tumor invasion, area of serosal invasion, and peritoneal metastasis. This procedure allows a rapid and reproducible analysis of large pools of samples. Although TRAP-ELISA appears to be a useful tool in detecting LNMM, its use in routine examinations is controversial due to the expensive property. Further research is needed to find the ideal marker with high accuracy and low cost to detect LNMM.

Despite of the satisfactory results, our study have several limitations. First, the present study included only a small sample data from a single institution, multicenter prospective trial that includes a larger number of patients will improve the performance. Second, due to the different proportions of patients with different stages of study, there will be some bias in the final result. Appropriate adjustment of the number of patients in different stages in future studies will reduce this bias. Finally, we only analyzed the MM from the level 2 station lymph nodes, the significance of the level 1 station LNMM is unknown, so all the resected lymph nodes should be investigated in the future.

In conclusion, our results demonstrated that the depth of invasion, TNM staging, lymphatic infiltration, and tumor differentiation were the independent risk factors associated with LNMM. We also revealed that LNMM is a clinically negative prognostic factor in pN0 GC patients. Therefore, for these high-risk patients with LNMM, more sensitive and accurate detection tools may be needed to obtain a precise diagnosis of MM, which is beneficial to develop individualized treatment plans and achieve favorable outcomes.

## Figures and Tables

**Table 1:** Clinicopathological factors of the patients associated with LNMM.

Clinicopathological factors, *n* (%)	LNMM		*P* value
	Yes	No	
Gender			0.059
Male	56 (77.8)	66 (66.0)	
Female	16 (22.2)	34 (34.0)	
Age			0.749
≤60	40 (55.6)	58 (58.0	
≥60	32 (44.4)	42 (42.0)	
Tumor size (cm)			0.031^∗^
≤2	6 (8.3)	25 (25.0)	
2~5	37 (51.4)	42 (42.0)	
≥5	29 (40.3)	33 (33.0)	
Tumor differentiation			0.032^∗^
High or moderate differentiation	13 (18.1)	34 (34.0)	
Low differentiation	59 (81.9)	66 (66.0)	
The depth of tumor invasion			0.001^∗^
T1	2 (2.8)	17 (17.0)	
T2	8 (11.1)	18 (18.0)	
T3	46 (63.9)	55 (55.0)	
T4	16 (22.2)	10 (10.0)	
Surgical approach			0.636
Total gastrectomy	49 (68.1)	70 (70.0)	
Distal gastrectomy	23 (31.9)	30 (30.0)	
General type			0.181
Topical type	23 (31.9)	42 (42.0)	
Infiltrative type	49 (68.1)	58 (58.0)	
Lauren classification			0.034^∗^
Diffusive type	51 (70.8)	53 (53.0)	
Intestinal type	21 (29.2)	47 (47.0)	
TNM staging			0.005^∗^
IA	2 (2.8)	17 (17.0)	
IB	8 (11.1)	18 (18.0)	
IIA	46 (63.9)	55 (55.0)	
IIB	11 (15.3)	8 (8.0)	
III	5 (6.9)	2 (2.0)	
Lymphatic infiltration			0.043^∗^
Yes	47 (65.3)	54 (54.0)	
No.	25 (34.7)	46 (46.0)	
Neural invasion			0.067
Yes	36 (50.0)	46 (46.0)	
No	36 (50.0)	54 (54.0)	

*n*: number; LNMM: lymph node micrometastasis; TNM: tumor node metastasis; *P* value was derived from the univariable association analyses between each characteristic; ^∗^*P* < 0.05.

**Table 2:** Multivariate analysis of clinicopathological factors associated with LNMM.

Variables	OR (95% CI)	*P* value
Tumor differentiation	1.266 (4.274-37.037)	0.013^∗^
Lymphatic infiltration	2.178 (1.635-6.327)	0.009^∗^
The depth of tumor invasion	3.755 (1.716-8.218)	0.004^∗^
Lauren classification	1.256 (0.513-3.631)	0.157
Tumor size	1.907 (0.706-5.156)	0.203
TNM staging	3.152 (1.547-7.589)	0.002^∗^

LNMM: lymph node micrometastasis; TNM: tumor node metastasis; OR: odd ratio; CI: confidence interval; ^∗^*P* < 0.05.

**Table 3:** Association of clinicopathological factors with a 5-year survival rate.

Clinicopathological factors	No. (%)	5-year survival rate (%)	*P* value
Gender			0.54
Male	122 (70.9)	60	
Female	50 (29.1)	54.9	
Age			0.95
≤60	98 (57.0)	58.1	
≥60	74 (43.0)	55.1	
Tumor size (cm)			0.01^∗^
≤2	31 (18.0)	96.8	
2~5	79 (45.9)	57	
≥5	62 (36.1)	35.5	
Tumor differentiation			0.00^∗^
High or moderate differentiation	47 (27.3)	87.5	
Low differentiation	125 (72.7)	47	
Level 2 LNMM			0.01^∗^
Positive	72 (41.9)	42	
Negative	100 (58.1)	76.4	
The depth of tumor invasion			0.01^∗^
T1	19 (11.1)	86.1	
T2	26 (15.1)	72.3	
T3	101 (58.7)	56.4	
T4	26 (15.1)	27.8	
Operation mode			0.07^∗^
Total gastrectomy	119 (69.2)	59.9	
Distal gastrectomy	53 (30.8)	37.5	
Neural invasion			0.42
Yes	37 (21.5)	51.4	
No	135 (78.5)	57.8	
Lauren classification			0.01^∗^
Diffusive type	104 (60.5)	48.1	
Intestinal type	68 (39.5)	69.1	
TNM staging			0.01^∗^
IA	19 (11.1)	86.1	
IB	26 (15.1)	72.3	
IIA	101 (58.8)	56.4	
IIB	19 (11.1)	30.1	
III	7 (9.9)	21.8	
Lymphatic infiltration			0.01^∗^
Yes	96 (55.8)	43.8	
No	76 (44.2)	72.4	

LNMM: lymph node micrometastasis; TNM: tumor node metastasis; ^∗^*P* < 0.05.

**Table 4:** Multivariate analysis of the prognostic factors associated with the survival.

Variables	SE	Wald	HR	95% CI	*P* value
Depth of tumor invasion	0.149	5.522	1.418	1.060-1.897	0.019
Level 2 station LNMM	0.286	10.082	2.476	1.415-4.334	0.001
Tumor size	0.230	8.779	1.978	1.260-3.106	0.003
TNM staging	0.150	4.473	1.375	1.024-1.846	0.034
Lauren classification	0.472	11.017	0.209	1.083-1.527	0.001
Lymphatic infiltration	0.272	15.219	2.893	1.697-4.934	0.000

LNMM: lymph node micrometastasis; TNM: tumor node metastasis; HR: hazard ratio; CI: confidence interval; SE: standard error.

## Data Availability

The datasets used and analyzed during the current study are available from the corresponding author on reasonable request.
